# Genetic Diversity of Meningococcal Serogroup B Vaccine Antigens among Carriage Isolates Collected from Students at Three Universities in the United States, 2015–2016

**DOI:** 10.1128/mBio.00855-21

**Published:** 2021-05-18

**Authors:** Henju Marjuki, How-Yi Chang, Nadav Topaz, Melissa J. Whaley, Jeni Vuong, Alexander Chen, Laurel T. Jenkins, Fang Hu, Susanna Schmink, Adam C. Retchless, Jennifer D. Thomas, Anna M. Acosta, Lucy A. McNamara, Heidi M. Soeters, Sarah Mbaeyi, Xin Wang

**Affiliations:** a Meningitis and Vaccine Preventable Diseases Branch, Division of Bacterial Diseases, National Center for Immunization and Respiratory Diseases, Centers for Disease Control and Prevention, Atlanta, Georgia, USA; b IHRC Inc., Contractor to Meningitis and Vaccine Preventable Diseases Branch, Division of Bacterial Diseases, National Center for Immunization and Respiratory Diseases, Centers for Disease Control and Prevention, Atlanta, Georgia, USA; c CDC Foundation Field Employee assigned to the Meningitis and Vaccine Preventable Diseases Branch, Division of Bacterial Diseases, National Center for Immunization and Respiratory Diseases, Centers for Disease Control and Prevention, Atlanta, Georgia, USA; GSK Vaccines; GSK Vaccines

**Keywords:** *Neisseria meningitidis*, carriage, serogroup B meningococcal vaccines, FHbp, NhbA, NadA, genetic diversity, whole-genome sequencing

## Abstract

Carriage evaluations were conducted during 2015 to 2016 at two U.S. universities in conjunction with the response to disease outbreaks caused by Neisseria meningitidis serogroup B and at a university where outbreak and response activities had not occurred. All eligible students at the two universities received the serogroup B meningococcal factor H binding protein vaccine (MenB-FHbp); 5.2% of students (181/3,509) at one university received MenB-4C. A total of 1,514 meningococcal carriage isolates were obtained from 8,905 oropharyngeal swabs from 7,001 unique participants. Whole-genome sequencing data were analyzed to understand MenB-FHbp’s impact on carriage and antigen genetic diversity and distribution. Of 1,422 isolates from carriers with known vaccination status (726 [51.0%] from MenB-FHbp-vaccinated, 42 [3.0%] from MenB-4C-vaccinated, and 654 [46.0%] from unvaccinated participants), 1,406 (98.9%) had intact *fHbp* alleles (716 from MenB-FHbp-vaccinated participants). Of 726 isolates from MenB-FHbp-vaccinated participants, 250 (34.4%) harbored FHbp peptides that may be covered by MenB-FHbp. Genogroup B was detected in 122/1,422 (8.6%) and 112/1,422 (7.9%) isolates from MenB-FHbp-vaccinated and unvaccinated participants, respectively. FHbp subfamily and peptide distributions between MenB-FHbp-vaccinated and unvaccinated participants were not statistically different. Eighteen of 161 MenB-FHbp-vaccinated repeat carriers (11.2%) acquired a new strain containing one or more new vaccine antigen peptides during multiple rounds of sample collection, which was not statistically different (*P* = 0.3176) from the unvaccinated repeat carriers (1/30; 3.3%). Our findings suggest that lack of MenB vaccine impact on carriage was not due to missing the intact *fHbp* gene; MenB-FHbp did not affect antigen genetic diversity and distribution during the study period.

## INTRODUCTION

Invasive meningococcal disease (IMD) caused by Neisseria meningitidis (the meningococcus) is a major public health concern (1). This bacterium can be asymptomatically carried as a commensal in the human nasopharyngeal mucosa. Exposure to meningococci can lead to either carriage or, less commonly, to IMD. Carriage prevalence varies among studies conducted in different populations and is age related, peaking during late adolescence in developed countries (2, 3). Risk factors, including behaviors linked with social mixing and smoking, have also been associated with carriage acquisition (3).

Six serogroups (A, B, C, W, X, and Y) cause most IMD cases worldwide, with B, C, and Y predominating in the United States (1, 4), and unencapsulated N. meningitidis (nongroupable, or NG) is commonly associated with carriage (5–8). Quadrivalent meningococcal conjugate vaccines confer protection against serogroups A, C, W, and Y and are routinely recommended for all U.S. adolescents aged 11 to 18 years as well as certain other individuals at increased meningococcal disease risk (9). Two protein-based serogroup B meningococcal (MenB) vaccines were licensed in the United States in 2014 to 2015 (10). MenB-FHbp (Trumenba; also known as bivalent rLP2086; Pfizer) contains two FHbp (factor H binding protein) peptides, one from subfamily A (A05) and one from subfamily B (B01) (Pfizer nomenclature) (10, 11), corresponding to peptides 3.45 and 1.55 (GlaxoSmithKline [GSK] nomenclature), respectively. MenB-4C (Bexsero [also known as 4CMenB]; GSK) has four components: FHbp B24 or 1.1, NhbA (neisserial heparin binding antigen) peptide 2 (p0002), NadA (neisserial adhesin A) peptide 3.8 (NadA-3.8), and outer membrane vesicle (OMV) from the N. meningitidis serogroup B strain NZ98/254 (derived from the MeNZB vaccine) with porin A variable region 2 (PorA-VR2) variant 4 as the major antigen (12, 13). In the United States, MenB vaccines are recommended for adolescents aged 16 to 23 years based on shared clinical decision making; they are also recommended for individuals aged 10 years or older who have increased risk for N. meningitidis serogroup B disease because of specific underlying conditions, a serogroup B outbreak, or occupational exposure as a microbiologist (14). Although licensed to protect against serogroup B disease, the vaccine antigens are also present in meningococci of other serogroups and may protect against non-B meningococcal disease (15, 16). While the long-term impact of MenB vaccines on meningococcal disease remains to be evaluated, collective evidence indicates that MenB vaccines do not have an impact on total or serogroup B carriage prevalence (7, 8, 17).

Outbreaks of serogroup B meningococcal disease were reported at 10 U.S. universities during 2013 to 2018 (18). Two outbreaks occurred in 2015 at universities in Rhode Island (RI-1) and Oregon (OR) (7, 8). MenB vaccination campaigns were implemented at each university for outbreak control. All eligible students at RI-1 received MenB-FHbp (8). While the majority of the eligible students at OR received MenB-FHbp, 5.2% of students received MenB-4C (7). Carriage evaluations conducted at the universities during 2015 to 2016 showed no decrease in meningococcal carriage following vaccination (7, 8). A third carriage evaluation was also conducted at a university near RI-1 (RI-2) as a reference population, where neither N. meningitidis serogroup B outbreaks nor MenB mass vaccination campaigns occurred (19). To understand the molecular mechanisms underlying the lack of impact of MenB-FHbp on carriage and the selective pressure of this vaccine on genetic diversity and prevalence of MenB vaccine antigens over time, we sequenced genomes of all carriage isolates collected from the three carriage evaluations and assessed potential changes in diversity and distribution of MenB vaccine antigens among vaccinated and unvaccinated students as well as changes in vaccine antigen profiles among students who participated in the carriage evaluations at multiple rounds of sample collection.

## RESULTS

### Genetic diversity and distribution of MenB vaccine antigens.

**FIG 1:**
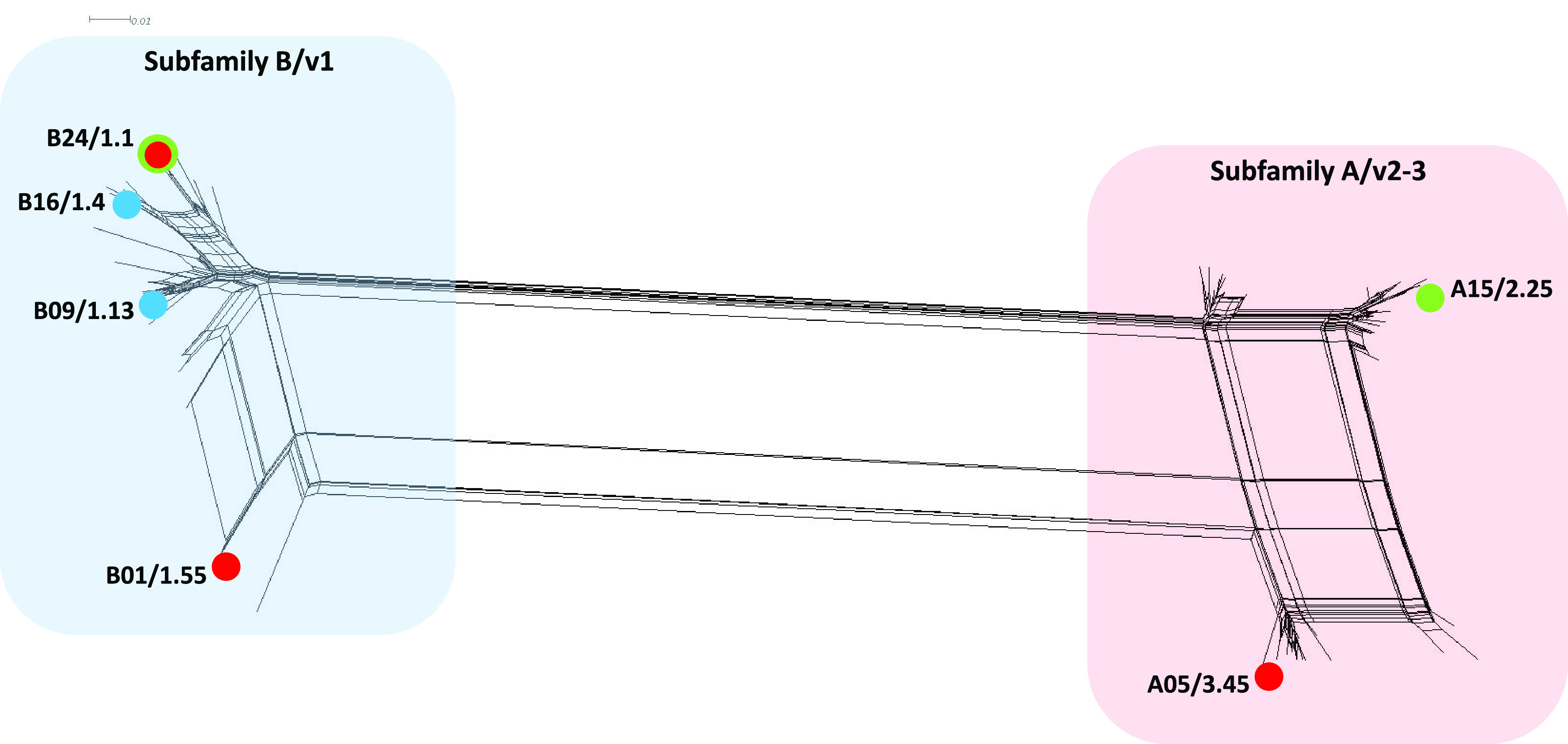
Phylogenetic analysis of unique peptide sequences of FHbp detected in carriage isolates in this study; all genogroups were included for this analysis. Peptides included in MenB-FHbp or MenB-4C vaccines are shown in red dots; the predominant peptides among carriage isolates are shown in blue dots. Prevalent peptides from invasive isolates obtained through domestic surveillance in the United States (via active bacterial core surveillance) between 2009 and 2014 (37) are shown in green dots.

Of the 1,337 isolates (540 from RI-1, 573 from OR, and 224 from RI-2) analyzed, 635 (47%) were either capsule null (*cnl*) or undetermined genogroup (see Table S1 in the supplemental material). The remaining 702 isolates belonged to the following capsular genogroups: E (384; 29%), B (225; 17%;), Y (33; 2.5%), C (24; 1.8%), Z (21; 1.6%), X (11; <1%), and W (4; <1%). Among the 1,337 carriage isolates, 1,326 (99.2%) isolates (532 from RI-1, 571 from OR, and 223 from RI-2) contained an intact *fHbp* allele; 11 (0.8%) isolates either lacked or contained a truncated *fHbp* allele (peptide not found; referred as FHbp negative) (Table 1). A higher number of carriage isolates contained FHbp peptides belonging to subfamily B/variant 1 (B/v1) (811/1,326; 61.2%) than subfamily A/variants 2/3 (A/v2-3) (515/1,326; 38.8%). Collectively, 61 unique FHbp peptides were identified: 37 (60.7%) from A/v2-3 and 24 (39.3%) from B/v1. FHbp B01/1.55, one of two FHbp antigens included in the MenB-FHbp vaccine (11), was not detected in any carriage isolate recovered in this study. FHbp A05/3.45, the other FHbp antigen included in MenB-FHbp (11), was detected in 23/1,326 (1.7%) isolates. FHbp B24/1.1 peptide, included in the MenB-4C vaccine (12), was detected in 40/1,326 (3.0%) isolates (Table 1). All FHbp peptides found among carriage isolates exhibited >82% amino acid sequence similarity to the FHbp peptides included in MenB vaccines (A05/3.45 or B24/1.1 and B01/1.55) within the same FHbp subfamily (Fig. 1).

**TABLE 1:** Distribution and diversity of FHbp among N. meningitidis carriage isolates collected from three U.S. universities, 2015 to 2016

Capsular genogroup	No. of isolates containing intact FHbp peptide^*a*^																				Total no. of intact FHbp peptide	No. with peptide not found	Total no.
	B24/1.1^*b*^	B.134/1.100	B06/1.12	B09/1.13	B30/1.224	B16/1.4	B168/1.544	B/v1 others (18 peptides)	A73/2.102	A19/2.16	A22/2.19	A07/2.21	A10/2.22	A12/2.24	A15/2.25	A106/2.27	A05/3.45^*c*^	A108/3.499	A103/3.94	A/v2-3 others (26 peptides)			
**B**	17			4	16	5		15		42	42	7	1	29			21	4	1	21	225		225
**C**				2		1		3		8				2	2			1		2	21	3	24
**E**	1		79	282		1	1	7		3				1	1			1	1	2	380	4	384
**W**										1		2			1						4		4
**X**				6							1				2				2		11		11
**Y**				1				3			1	2		1	20					5	33		33
**Z**				1						4			11		5						21		21
**UD**	6		1	1				2		14	1		1	8	2	18	2	1		6	63	2	65
***cnl***	16	26		18		277	9	10	46	3	10	9		22				30	69	23	568	2	570
	
**Total**	40	26	80	315	16	284	10	40	46	75	55	20	13	63	33	18	23	37	71	61	1,326	11	1,337

a Both Pfizer (MenB-FHbp) and GSK (MenB-4C) nomenclatures are shown. Unless included in the MenB vaccines, only major peptide variants of each antigen (detected in ≥10 isolates) are shown. Intact FHbp peptide was found in 532 isolates from RI-1, 571 isolates from OR, and 223 isolates from RI-2. Abbreviations: UD, undetermined (unable to identify serogroup-specific genes); *cnl*, capsule null locus.

b Included in MenB-4C vaccine.

c Included in MenB-FHbp vaccine (the other antigen, FHbp B01/1.55, was not present in this collection).

10.1128/mBio.00855-21.2 TABLE S1 Number of carriage isolates by genogroup. Download Table S1, PDF file, 0.03 MB. Copyright © 2021 Marjuki et al. 2021 Marjuki et al. https://creativecommons.org/licenses/by/4.0/ This content is distributed under the terms of the Creative Commons Attribution 4.0 International license.

All genogroup B isolates (225/1,326; 17.0%) contained an intact *fHbp* allele, with 28 unique FHbp peptides (19 belonging to A/v2-3 and nine to B/v1) being detected, and had higher sequencing diversity than other genogroups. FHbp B/v1 peptides were detected among 57/225 (25.3%) genogroup B isolates; all remaining genogroup B isolates contained FHbp A/v2-3. FHbp A05/3.45 was detected in 21/225 (9.3%) genogroup B isolates, and FHbp B24/1.1 was detected in 17/225 (7.6%) genogroup B isolates (Table 1).

An intact *nhbA* allele was detected in 1,331 (99.6%) carriage isolates, with 90 unique NhbA peptides being identified (Table S2). NhbA p0002, the peptide included in the MenB-4C vaccine (12), was detected in 71/1,331 (5.3%) carriage isolates, of which 31 (43.7%) were genogroup B isolates. NhbA peptides identified among carriage isolates in this study showed >67% sequence similarity to the MenB-4C component NhbA p0002 (Fig. S1A).

10.1128/mBio.00855-21.1 FIG S1 Phylogenetic analysis of unique peptide sequences of NhbA and NadA. Download FIG S1, PDF file, 0.2 MB. Copyright © 2021 Marjuki et al. 2021 Marjuki et al. https://creativecommons.org/licenses/by/4.0/ This content is distributed under the terms of the Creative Commons Attribution 4.0 International license.

10.1128/mBio.00855-21.3 TABLE S2 Distribution and diversity of NhbA among N. meningitidis carriage isolates. Download Table S2, PDF file, 0.1 MB. Copyright © 2021 Marjuki et al. 2021 Marjuki et al. https://creativecommons.org/licenses/by/4.0/ This content is distributed under the terms of the Creative Commons Attribution 4.0 International license.

Only 93 (7.0%) carriage isolates had intact *nadA* alleles, with 12 unique NadA peptides being identified (Table S3). NadA-3.8, included in the MenB-4C vaccine (12), was detected in two isolates: one in genogroup B and one in genogroup Y. NadA-1 and NadA-2/3 identified among carriage isolates showed >66% sequence similarity to NadA-3.8 in MenB-4C, while NadA-4/5 and NadA-6 showed 37% sequence similarity to NadA-3.8 (Fig. S1B).

10.1128/mBio.00855-21.4 TABLE S3 Distribution and diversity of NadA among N. meningitidis carriage isolates. Download Table S3, PDF file, 0.09 MB. Copyright © 2021 Marjuki et al. 2021 Marjuki et al. https://creativecommons.org/licenses/by/4.0/ This content is distributed under the terms of the Creative Commons Attribution 4.0 International license.

Overall, there were 1,320 (98.7%) isolates with both *fHbp* and *nhbA* intact. Ninety-two (6.9%) carriage isolates contained all three intact *fHbp*, *nhbA*, and *nadA* genes, of which 44 (47.8%) belonged to capsular genogroup B.

Among 1,337 carriage isolates, 1,316 (98.4%) harbored intact *porA* alleles that belonged to 207 PorA types, and 9/1,316 (<1%) (seven genogroup B, one capsule null, and one undetermined genogroup) contained PorA-VR2 variant 4, included in the MenB-4C vaccine (13).

### FHbp subfamily distribution among vaccinated and unvaccinated participants.

MenB-FHbp was provided to all eligible students at RI-1 and most eligible students at OR; MenB-4C was provided to 5.2% (181 of 3,509) of students at OR. A total of 1,422 N. meningitidis isolates (646 from RI-1, 528 from OR, and 248 from RI-2) were recovered from participants with known vaccination status (Table S4), with 726 (51.0%) from MenB-FHbp-vaccinated participants, 42 (3.0%) from MenB-4C-vaccinated participants, and 654 (46.0%) from unvaccinated participants. Of the 238/1,422 (16.7%) genogroup B isolates, 122/1,422 (8.6%) and 4/1,422 (0.3%) were from MenB-FHbp- and MenB-4C-vaccinated participants, respectively, and 112/1,422 (7.9%) were from unvaccinated participants.

10.1128/mBio.00855-21.5 TABLE S4 Number of isolates from participants with known MenB vaccine records. Download Table S4, PDF file, 0.03 MB. Copyright © 2021 Marjuki et al. 2021 Marjuki et al. https://creativecommons.org/licenses/by/4.0/ This content is distributed under the terms of the Creative Commons Attribution 4.0 International license.

Among the 1,422 isolates, 1,406 (98.9%) had intact *fHbp* alleles: 716/1,422 (50.4%) from MenB-FHbp-vaccinated participants, 42/1,422 (3.0%) from MenB-4C-vaccinated participants, and 648/1,422 (45.6%) from unvaccinated participants. The distribution of FHbp subfamilies in MenB-FHbp-vaccinated and unvaccinated groups was analyzed for all three universities as well as each university (Table 2). Overall, for all three universities, higher proportions of isolates contained FHbp B/v1 than FHbp A/v2-3 among all or non-B genogroups within both vaccinated and unvaccinated groups. In contrast, a higher proportion of genogroup B isolates contained FHbp A/v2-3 than B/v1 within both groups. There was no significant difference in FHbp subfamily distribution between vaccinated and unvaccinated groups among all genogroups, B genogroup, and non-B genogroups observed for RI-1 and OR. At OR, while the *P* values for FHbp subfamily distribution between vaccinated and unvaccinated participants among genogroup B and non-B genogroups were <0.05, there was no statistically significant difference between the two groups after Bonferroni correction.

**TABLE 2:** FHbp subfamily among carriage isolates, stratified by university and MenB-FHbp vaccination status

University	Capsular genogroup	Vaccination status	Total no. of isolates^*a*^	FHbp a/v2-3 [no. (%)]	FHbp B/v1 [no. (%)]	FHbp negative	Fisher exact *P* value^*b*^
RI-1	All	Unvaccinated	195	59 (30.3)	132 (67.7)	4 (2.1)	1
	All	Vaccinated	451	137 (30.4)	305 (67.6)	9 (2.0)	
	B	Unvaccinated	41	31 (75.6)	10 (24.4)	0 (0.0)	0.1146
	B	Vaccinated	89	54 (60.7)	35 (39.3)	0 (0.0)	
	Non-B	Unvaccinated	154	28 (18.2)	122 (79.2)	4 (2.6)	0.2425
	Non-B	Vaccinated	362	82 (22.7)	270 (74.6)	9 (2.5)	
OR	All	Unvaccinated	211	91 (43.1)	119 (56.4)	1 (0.5)	0.0545
	All	Vaccinated	275	99 (36.0)	175 (63.6)	1 (0.4)	
	B	Unvaccinated	29	24 (82.8)	5 (17.2)	0 (0.0)	0.0133*
	B	Vaccinated	33	33 (100)	0	0 (0.0)	
	Non-B	Unvaccinated	182	67 (36.8)	114 (62.6)	1 (0.6)	0.0169*
	Non-B	Vaccinated	242	66 (27.3)	175 (72.3)	1 (0.4)	
RI-2	All	Unvaccinated	248	142 (57.3)	105 (42.3)	1 (0.4)	NA
	B	Unvaccinated	42	32 (76.2)	10 (23.8)	0 (0.0)	
	Non-B	Unvaccinated	206	110 (53.4)	95 (46.1)	1 (0.5)	

a Forty-two isolates from MenB-4C participants were excluded for this analysis, resulting in a total of 1,380 isolates from unvaccinated and MenB-FHbp-vaccinated participants from each university with intact or missing *fHbp* allele. FHbp-negative isolates were excluded from the statistical test.

b An asterisk indicates not significant after Bonferroni correction of *n* = 6 tests. NA, not applicable.

### FHbp peptides distribution and predicted strain coverage among vaccinated and unvaccinated participants.

**FIG 2:**
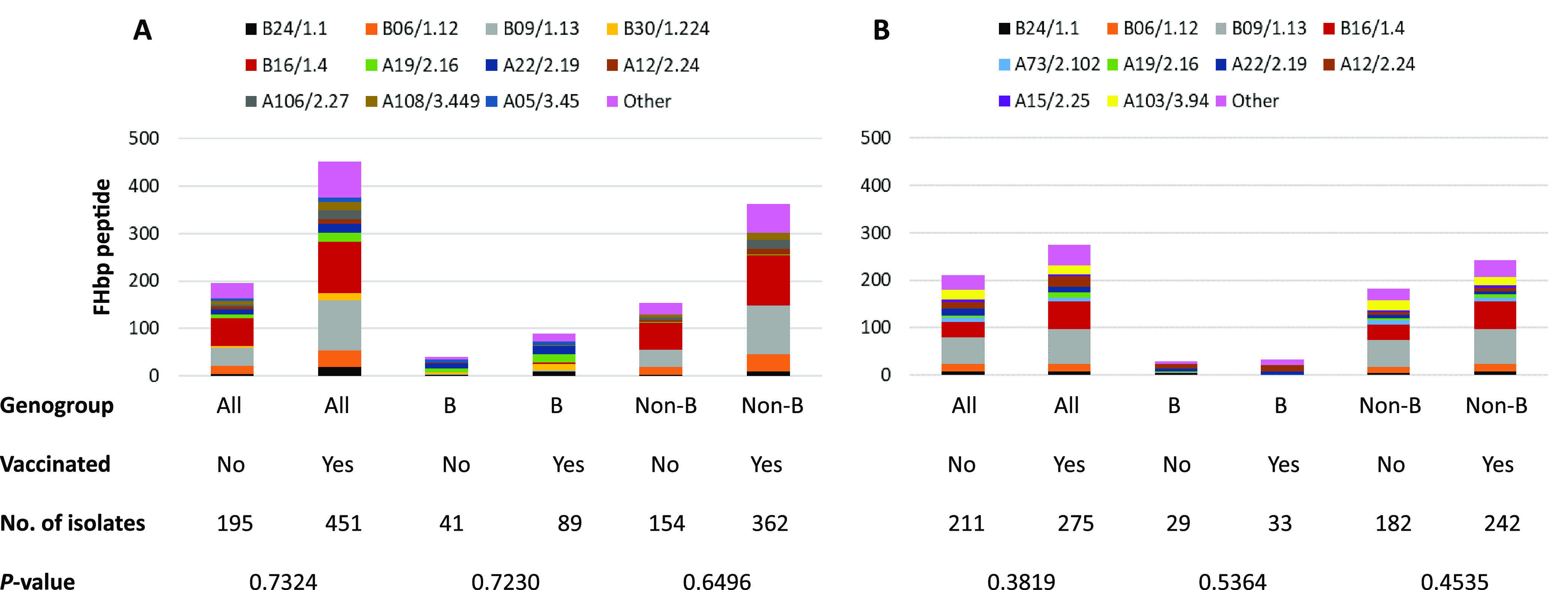
Predominant FHbp peptides among carriage isolates, stratified by university (A, RI-1; B, OR) and vaccination status. All isolates, except 42 from MenB-4C participants, were included for the analysis.

The distribution of FHbp peptides was assessed among carriage isolates from MenB-FHbp-vaccinated and unvaccinated participants at RI-1 and OR (Fig. 2). Overall, isolates containing FHbp B09/1.13 and B16/1.4 peptides were predominant among MenB-FHbp-vaccinated groups (215/646; 33.3% at RI-1 and 133/528; 25.2% at OR) and unvaccinated groups (95/646; 14.7% at RI-1 and 90/528; 17.1% at OR). Only eight genogroup B isolates contained either FHbp B09/1.13 or B16/1.4, six from MenB-FHbp-vaccinated (all at RI-1) and 2 from unvaccinated (one each at RI-1 and OR) participants. A small proportion of isolates either lacked the *fHbp* allele or contained a truncated *fHbp* allele, including nine isolates from MenB-FHbp-vaccinated participants and four isolates from unvaccinated participants at RI-1 and two isolates from OR (one each from MenB-FHbp-vaccinated and unvaccinated participants). There was no statistically significant difference in FHbp peptide distributions between vaccinated and unvaccinated participants among genogroup B isolates and non-B genogroups from each university.

While FHbp B01/1.55 was not detected in any isolate in this study, FHbp A05/3.45 was present in 12 of 726 (1.7%) isolates (11 genogroup B and one undetermined genogroup) from MenB-FHbp-vaccinated participants (nine from RI-1 and three from OR), all of which were from six MenB-FHbp-vaccinated participants (each received either one or two vaccine doses). Other FHbp peptides (A04/3.180, A22/2.19, B09/1.13, B24/1.1, and B44/1.15), shown to be covered by MenB-FHbp (20–24), were detected in 1/726 (0.1%), 29/726 (4.0%), 180/726 (24.8%), 26/726 (3.6%), and 2/726 (0.3%) isolates from MenB-FHbp-vaccinated participants, respectively. A total of 84 isolates containing FHbp that were covered by MenB-FHbp belonged to genogroup B isolates: 46 from MenB-FHbp-vaccinated participants (37 from RI-1 and 9 from OR) and 38 from unvaccinated participants (22 from RI-1 and 16 from OR).

### Vaccination impact on carriage acquisition in repeat participants.

Of 194 repeat carriers, 161 (83.0%) received MenB-FHbp, 3 (1.5%) received MenB-4C, and 30 (15.5%) did not receive a vaccine. Among 161 MenB-FHbp-vaccinated repeat carriers, 143 (88.8%) had 302 isolates that retained the same strain genotype and 18 (11.2%) had 40 isolates that acquired a new strain genotype (Table S5). Among the 30 unvaccinated repeat carriers, 29 (96.7%) had 58 isolates that retained the same strain genotype, and one (3.3%) from RI-2 had two isolates that acquired a new strain genotype. The difference between vaccinated and unvaccinated repeat carriers who acquired a new strain was not statistically significant (*P* = 0.3176).

10.1128/mBio.00855-21.6 TABLE S5 Sequence typing profiles of N. meningitidis isolates recovered from repeat carriers who carried new strains during the carriage evaluation period. Download Table S5, PDF file, 0.1 MB. Copyright © 2021 Marjuki et al. 2021 Marjuki et al. https://creativecommons.org/licenses/by/4.0/ This content is distributed under the terms of the Creative Commons Attribution 4.0 International license.

Of the 342 isolates (53 genogroup B isolates: 44 from those retaining the same strain genotype, 9 from those acquiring a new strain genotype) carried by MenB-FHbp-vaccinated repeat carriers, 32 unique FHbp peptides were detected, with B16/1.4 (96/342; 28.1%) being the most prevalent, followed by B09/1.13 (68/342; 19.9%). Within the 302 isolates that retained the same strain genotype, six each (2.0%) harbored FHbp A05/3.45 or B24/1.1. Among the 60 isolates (10 genogroup B isolates) from unvaccinated participants, 13 unique FHbp peptides were detected, with B16/1.4 (16/60; 26.7%) being most prevalent. There was a statistical difference in FHbp peptide distribution between vaccinated and unvaccinated repeat carriers among all isolates (*P* < 0.0001) and genogroup B isolates (*P* = 0.0225). Additionally, of the 42 isolates that acquired a new strain genotype, the FHbp peptide prevalence between the detected strains in the previous round and newly acquired strains in the following round changed from B09/1.13 (seven; 16.7%) to B16/1.4 (three; 7.1%) and from p0010 and p0024 (five each; 11.9%) to p0021 (seven; 16.7%) for NhbA (Table S5).

## DISCUSSION

Analysis of whole-genome sequencing data has allowed us to understand MenB vaccine antigens and assess the impact of MenB-FHbp on their genetic diversity and distribution in U.S. meningococcal carriage. Almost all carriage isolates (99%) contained an intact *fHbp* gene, with 61% belonging to FHbp B/v1 peptides. These findings are in contrast to a previous study showing a higher proportion of FHbp A/v2-3 peptides among carriage isolates (25), but the proportion of genogroup B isolates in this prior study was greater than that found in our study. Similarly, the majority of carriage isolates (>99%) in our study contain an intact *nhbA* gene, with 99% of isolates harboring both *fHbp* and *nhbA*. In contrast, the *nadA* gene was detected in only a small proportion (7%) of carriage isolates. We found an equal proportion of carriage isolates with an intact *fHbp* gene recovered from vaccinated (95% received MenB-FHbp) and unvaccinated participants. Statistical analysis showed no significant difference in the distribution of FHbp subfamilies or peptides between vaccinated and unvaccinated populations, suggesting that the vaccine does not impact the overall FHbp diversity and distribution.

Comparative whole-genome analyses provide evidence of ongoing genetic shift and gene loss in Bordetella pertussis following the introduction of pertussis vaccines, possibly due to vaccine selective pressure (26). Our data suggest that MenB-FHbp vaccination did not exert significant selective pressure on MenB vaccine antigens in our study. We found that 63% of carriage isolates lacking or containing a truncated form of the *fHbp* allele were from MenB-FHbp-vaccinated participants. However, whether MenB-FHbp administration exerts a long-term impact similar to that observed with pertussis vaccines warrants further investigations. Most repeat carriers received MenB-FHbp. Almost all strains recovered from MenB-FHbp-vaccinated and unvaccinated repeat carriers retained the same vaccine antigen peptides. Only a small percentage of carriage isolates recovered from the vaccinated repeat carriers acquired a new strain with different vaccine antigen peptides. Among isolates from the repeat carriers, we observed significant differences in FHbp peptide distribution between vaccinated and unvaccinated groups. However, it could be due to the much higher proportion of isolates from MenB-FHbp-vaccinated than from unvaccinated repeat carriers.

Previous analyses indicated that the meningococcal carriage rate remained unchanged following vaccination campaigns at RI-1 and OR, where the majority of participants received MenB-FHbp (7, 8). However, it remains unclear whether vaccine-induced antibodies will affect the overall meningococcal or serogroup B carriage long term. Clinical studies conducted in healthy individuals vaccinated with MenB-FHbp demonstrate a robust induction of bactericidal antibodies by the vaccine against meningococcal strains expressing vaccine-homologous (A05/3.45) and -heterologous (A04/3.180, A17/2.49, A22/2.19, A56/3.187, B02/1.87, B09/1.13, B24/1.1, or B44/1.15) FHbp peptides, as determined by bactericidal activity assay using human complement (hSBA), a method to predict vaccine coverage (20–24). In our study, FHbp A04/3.180, A05/3.45, A22/2.19, B09/1.13, B24/1.1, and B44/1.15 were detected in 34% of isolates from MenB-FHbp-vaccinated participants, suggesting that these strains are covered by the vaccine if FHbp is sufficiently expressed on the surface of these strains.

While the genomic data in this study showed that a high proportion of carriage isolates harbored an intact *fHbp* gene, FHbp surface expression has not been evaluated among these isolates. The antigen surface expression level and its accessibility to bactericidal antibodies are essential factors that can impact vaccine effectiveness (27–29). The genetic diversity and expression of FHbp may affect the bactericidal activity of FHbp-specific antibodies. It is conceivable that a lack of vaccine impact on carriage is due to the low level of antigen expression. A carriage study conducted in France among individuals aged 1 to 25 years showed that FHbp expression was significantly lower in carriage isolates than in invasive isolates; 32% of all carriage isolates tested had no detectable FHbp (25). Further investigations on the level of vaccine antigen surface expression in the carriage isolates from our study may shed light on mechanisms underlying the lack of impact of MenB vaccines on carriage. While induction of mucosal antibody responses has been demonstrated for meningococcal serogroup C polysaccharide-conjugate vaccines, thereby reducing the carriage and improving the level of herd protection (30–32), this effect has not been confidently demonstrated for MenB vaccines. Prior research suggests that a mucosal mode of administration is required to achieve an adequate immune response within the mucosal environment using MenB vaccines (33–35). Finally, although MenB vaccine-induced bactericidal activity is a potential surrogate marker for immunity after vaccination against invasive meningococcal diseases, its correlation with meningococcal carriage remains unclear.

We recently showed that most of the carriage isolates belong to CC198 and CC1157, with a very low proportion of carriage isolates in this study belonging to hyperinvasive lineages such as CC32, CC41/44, and CC11 (36), consistent with a previous report (25). Therefore, MenB vaccine antigens that are typically found in isolates of hyperinvasive lineages were detected in only a small proportion of carriage isolates. Previous studies indicated that 56 to 60% of invasive serogroup B isolates collected during 2000 to 2014 in the United States contain FHbp B/v1, with FHbp B24/1.1 included in MenB-4C being the most prevalent (33 to 34%) (37, 38); this particular peptide was also present in the meningococcal outbreak strains from RI-1 (ST-9069) and OR (ST-32/CC32). In our study, FHbp B/v1 peptides were carried by 25% of genogroup B carriage isolates, with FHbp B24/1.1 being detected in 8% of genogroup B isolates. FHbp A05/3.45, included in MenB-FHbp, was detected in 9% of genogroup B isolates, while FHbp B01/1.55 was absent from all carriage isolates analyzed in this study. Similarly, these two FHbp peptides were either absent or present at a low prevalence among invasive isolates (37). Additionally, NhbA p0002, included in MenB-4C, showed greater prevalence among invasive N. meningitidis serogroup B isolates (9 to 11%) (37, 38) than among genogroup B carriage isolates (2%).

Overall, similar FHbp peptide distributions were observed among carriage isolates from both vaccinated and unvaccinated participants during the study period. Additional investigations over a longer period are warranted to adequately evaluate the vaccine impact on carriage.

## MATERIALS AND METHODS

### Data collection.

Isolates included in this study were collected during February 2015 to March 2016 from meningococcal carriage evaluations at three U.S. universities (RI-1, RI-2, and OR) following vaccination using standardized methods as previously described (7, 8, 19). Briefly, four carriage evaluation rounds were conducted in conjunction with mass vaccination campaigns at RI-1 (February, April, and September 2015 and March 2016) and OR (March, May, and October 2015 and February 2016); two carriage evaluation rounds (March and April 2015) were conducted at RI-2, which is located in the same city as RI-1 and where no N. meningitidis serogroup B outbreak and response activities occurred. A total of 1,514 meningococcal isolates (650 from RI-1, 616 from OR, and 248 from RI-2) were recovered from 8,905 oropharyngeal swabs collected from 7,001 unique individuals (2,014 from RI-1, 3,509 from OR, and 1,478 from RI-2). N. meningitidis species was determined using real-time PCR targeting *sodC* (39), and serogroup was determined using slide agglutination serogrouping (SASG) (40, 41). A total of 1,587 individuals (615 from RI-1, 613 from OR, and 359 from RI-2) participated in multiple evaluation rounds (repeat participants); 348 were identified to be N. meningitidis carriers, with 154 carrying N. meningitidis in only one round and 194 carrying N. meningitidis in at least 2 rounds (repeat carriers).

These evaluations were considered public health evaluations and did not require CDC institutional review for human subjects’ protection. Both evaluations were covered under project determination numbers 2015–6436 and 2015–6442 for “Evaluation of Neisseria meningitidis serogroup B carriage in institutional settings during an outbreak.”

### Molecular characterization.

Bacterial genomes of all 1,514 confirmed meningococcal isolates were further characterized with whole-genome sequencing using Illumina platforms (HiSeq2500 or MiSeq; San Diego, CA). Illumina reads were trimmed with cutadapt (42) to remove adaptor sequences and reads below a quality score of 28 (Q28) and 75 bp. *De novo* short-read assembly was carried out using SPAdes 3.7.0 (43) with the “careful” option. The capsular genogroup of each isolate was determined based on serogroup-specific genes (44); isolates that contained capsule genes but lacked any identifiable serogroup-specific capsule gene were deemed to have an “undetermined genogroup.” Isolates that lack the whole-capsule locus are defined as capsule null (*cnl*) (44). Genome sequence assemblies were used in subsequent BLAST searches (45) against the PubMLST *Neisseria* allele database (46) to determine clonal complex (CC)/sequence type (ST), MenB vaccine antigens (FHbp, NhbA, and NadA), and fine typing peptides (FetA and PorA) (47). All unique peptides of MenB vaccine antigens, including PorA, were assigned PubMLST peptide allele identifiers (IDs) as described previously (12, 48, 49).

### Data analysis.

While 1,514 carriage isolates were obtained, only one isolate from each participant who carried the same meningococcal strain in more than one round (repeat carriers) was included in the analysis to assess the MenB vaccine antigen diversity and distribution among the carriage isolates circulating in the three universities, resulting in a total of 1,337 isolates.

To compare MenB vaccine antigen distribution between vaccinated (received ≥1 dose of MenB-FHbp or MenB-4C 14 days prior to sample collection) and unvaccinated participants, all isolates from repeat carriers were included even if they appeared to be the same strain genotype (CC:ST:PorA:FetA); however, participants with unknown MenB vaccination records were excluded from the analysis (92 isolates from RI-1 and OR), resulting in a total of 1,422 carriage isolates. For phylogenetic analysis of MenB vaccine antigens, multiple-sequence alignment of peptides was generated by ClustalW (50) with CLC Genomics Workbench 7. Networks were created by SplitsTree, v 4.0 (51), with default parameters.

### Statistical analysis.

Statistical analysis was performed using SAS version 9.4 (SAS Institute, Cary, NC). Fisher’s exact test was used to assess changes in the distribution of FHbp subfamilies and peptides in isolates recovered from vaccinated and unvaccinated participants, stratified by university and whether the isolate was N. meningitidis serogroup B or non-N. meningitidis serogroup B. Isolates negative for both subfamilies A and B were excluded from this test due to being a low proportion (0.9%) of the overall isolate collection. Chi-squared goodness-of-fit tests were used to test for changes in distribution among N. meningitidis serogroup B and non-N. meningitidis serogroup B isolates. A Bonferroni correction was applied to correct for multiple comparisons. To assess proportions between vaccinated and unvaccinated repeat carriers who acquired a new meningococcal strain, a Fisher’s exact test was performed; FHbp peptide distributions in all isolates and genogroup B isolates among these groups were analyzed using a likelihood ratio chi-squared test.

### Data availability.

Sequence reads are available under NCBI BioProject no. PRJNA533315.
