# Supplementary material for: Genetic Diversity of Meningococcal Serogroup B Vaccine Antigens among Carriage Isolates Collected from Students at Three Universities in the United States, 2015–2016
Source: mBio. 2021 May 18;12(3):e00855-21. doi: 10.1128/mBio.00855-21 (PMC8262942; doi:10.1128/mBio.00855-21)
Supplement: TABLE S1 [file mbio.00855-21-st001.pdf]

Table S1: Number of carriage isolates by genogroup.

**University**	**Genogroup**									
	B	C	E	W	X	Y	Z	UD	*cnl*	Total
RI-1	113	11	154	2	6	10	4	29	211	540
OR	74	8	204	2	2	11	8	22	242	573
RI-2	38	5	26	0	3	12	9	14	117	224
Total	225	24	384	4	11	33	21	65	570	1,337

Abbreviations: UD, undetermined (unable to identify serogroup-specific genes); *cnl*, capsule null locus
