# Supplementary material for: Genetic Diversity of Meningococcal Serogroup B Vaccine Antigens among Carriage Isolates Collected from Students at Three Universities in the United States, 2015–2016
Source: mBio. 2021 May 18;12(3):e00855-21. doi: 10.1128/mBio.00855-21 (PMC8262942; doi:10.1128/mBio.00855-21)

Fig. S1. Phylogenetic analysis of unique peptide sequences of (A) NhbA and (B) NadA detected in carriage isolates in this study; all genogroups were included for this analysis. Peptides included in MenB-4C are shown in red dots; the predominant peptides among carriage isolates are shown in blue dots. Prevalent peptides from invasive isolates obtained through domestic surveillance in the United States (via Active Bacterial Core surveillance) between 2009-2014 are shown in green dots.

A)

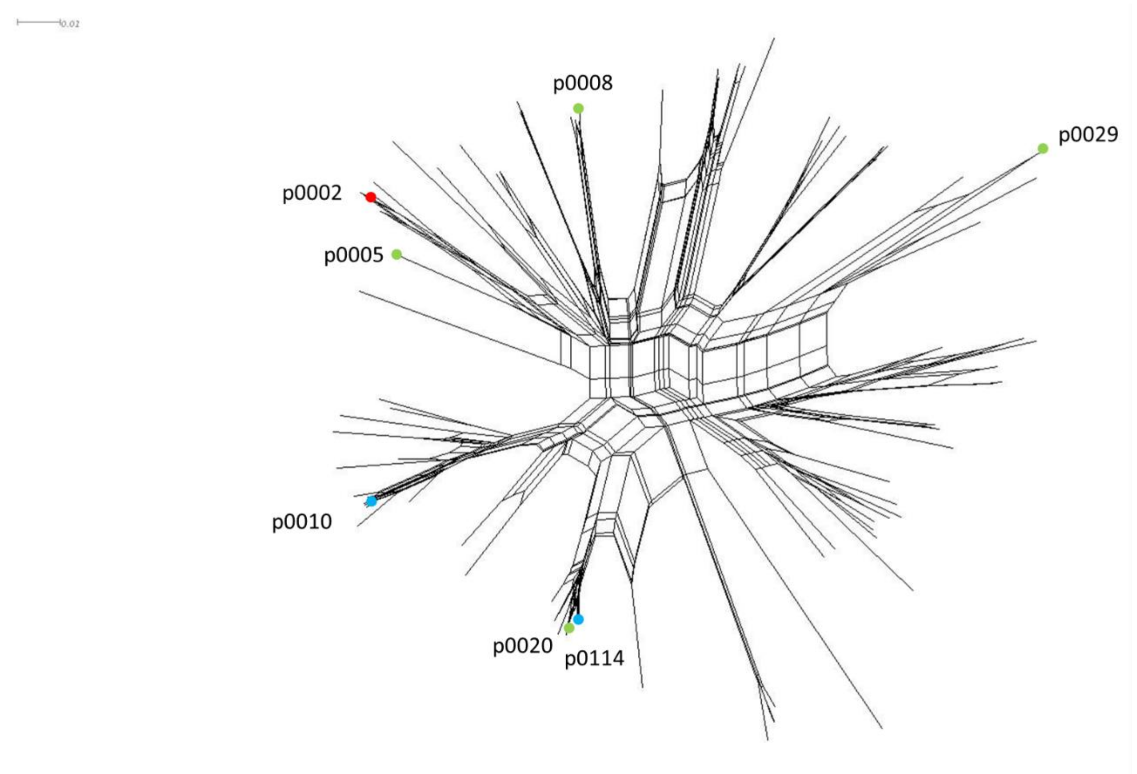

B)

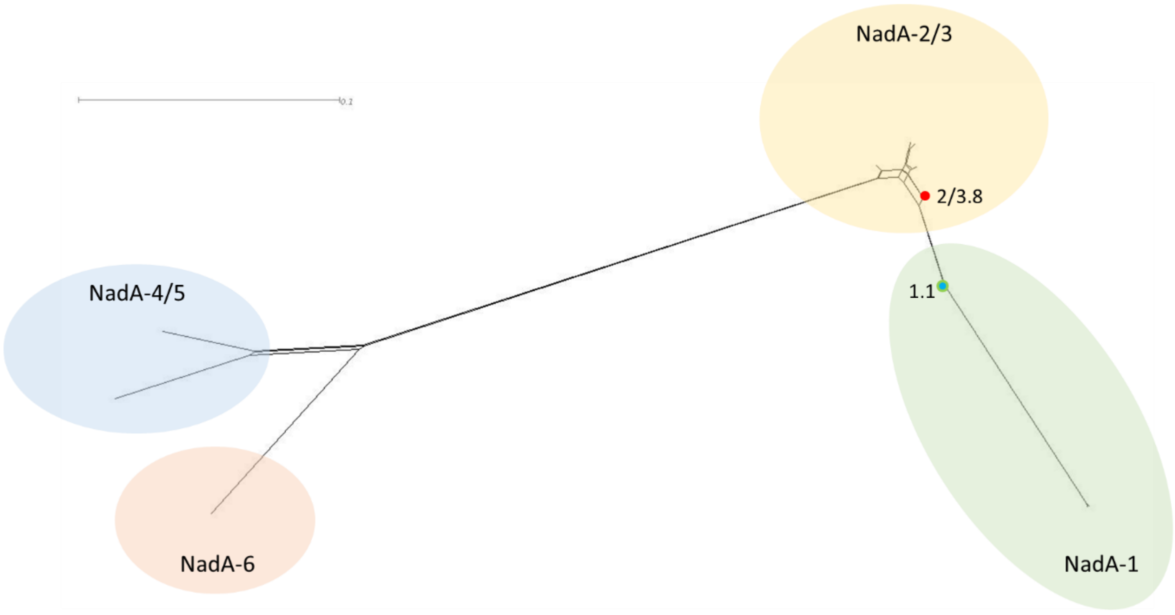

Supplement: FIG S1 [file mbio.00855-21-sf001.pdf]
