# Supplementary material for: Genetic Diversity of Meningococcal Serogroup B Vaccine Antigens among Carriage Isolates Collected from Students at Three Universities in the United States, 2015–2016
Source: mBio. 2021 May 18;12(3):e00855-21. doi: 10.1128/mBio.00855-21 (PMC8262942; doi:10.1128/mBio.00855-21)
Supplement: TABLE S2 [file mbio.00855-21-st002.pdf]

Table S2. Distribution and diversity of NhbA among *N. meningitidis* carriage isolates collected from three U.S. universities, 2015–2016.

**Capsular Genogroup**	**Intact NhbA peptide**[a]																**Total intact NhbA peptide**	**Peptide not found**	**Total**
	p0002[b]	p0003	p0006	p0007	p0010	p0016	p0018	p0020	p0021	p0024	p0058	p0092	p0114	p0145	p0601	Others (75 peptides)			
**B**	31	35	1		24		20	10	42	3		1	2			56	225		225
**C**		1							20							3	24		24
**E**			82		50			1		82		2	144			21	382	2	384
**W**								2								2	4		4
**X**													5			6	11		11
**Y**			2	18	3								2			8	33		33
**Z**										4			1		12	4	21		21
**UD**	1	8	1	1	1		1	5	29							18	65		65
cnl	39				260	55		19	39	2	49	19	12	19		53	566	4	570
**Total**	71	44	86	19	338	55	21	37	130	91	49	22	166	19	12	171	1,331	6	1,337

Abbreviations: UD, undetermined (unable to identify serogroup-specific genes); *cnl*, capsule null locus.
[a] Intact NhbA peptide was found in 535 isolates from RI-1, 572 isolates from OR, and 224 isolates from RI-2. Unless included in the MenB vaccines, only major peptide variants of each antigen (detected in ≥10 isolates) are shown.
[b] Included in MenB-4C vaccine.
