# Supplementary material for: Genetic Diversity of Meningococcal Serogroup B Vaccine Antigens among Carriage Isolates Collected from Students at Three Universities in the United States, 2015–2016
Source: mBio. 2021 May 18;12(3):e00855-21. doi: 10.1128/mBio.00855-21 (PMC8262942; doi:10.1128/mBio.00855-21)
Supplement: TABLE S3 [file mbio.00855-21-st003.pdf]

Table S3. Distribution and diversity of NadA among *N. meningitidis* carriage isolates collected from three U.S. universities, 2015–2016.

**Capsular Genogroup**	**Intact NadA peptide**[a]				**Total intact NadA peptide**	**Peptide not found**	**Total**
	NadA-1.1	NadA-1.100	NadA-3.8[b]	Others (9 peptides)			
**B**	23	17	1	3	44	181	225
**C**	2				2	22	24
**E**				9	9	375	384
**W**						4	4
**X**				8	8	3	11
**Y**			1	4	5	28	33
**Z**						21	21
**UD**	7				7	58	65
cnl	15			3	18	552	570
**Total**	47	17	2	27	93	1,244	1,337

Abbreviations: UD, undetermined (unable to identify serogroup-specific genes); *cnl*, capsule null locus.

[a] Intact NadA peptide was found in 58 isolates from RI-1, 26 isolates from OR, and 9 isolates from RI-2. Unless included in the MenB vaccines, only major peptide variants of each antigen (detected in ≥10 isolates) are shown.

[b] Included in MenB-4C vaccine.
