# Supplementary material for: Genetic Diversity of Meningococcal Serogroup B Vaccine Antigens among Carriage Isolates Collected from Students at Three Universities in the United States, 2015–2016
Source: mBio. 2021 May 18;12(3):e00855-21. doi: 10.1128/mBio.00855-21 (PMC8262942; doi:10.1128/mBio.00855-21)
Supplement: TABLE S4 [file mbio.00855-21-st004.pdf]

Table S4: Number of isolates from participants with known MenB vaccine records.

		RI-1	OR	RI-2
Count (%)		646[a]/650 (99.4%)	528 [a] /616 (85.7%)	248 (100%)
Unvaccinated		195 (30.2%)	211 (40%)	248
Vaccinated		451 (69.8%)<br>MenB-FHbp: 451 (100%)	317 (60%)<br>MenB-FHbp: 275 (86.8%)<br>MenB-4C: 42 (13.2%)	N/A
MenB-FHbp doses	1	233	181	N/A
	2	182	124	N/A
	3	36	12	N/A
MenB-4C doses	1	0	7	N/A
	2	0	35	N/A

Abbreviation: N/A, not applicable (no vaccination campaign occurred at this site).

[a] Four isolates from RI-1 and 88 isolates from OR were excluded from the analysis due to the lack of MenB vaccine records for the participant, resulting in a total of 1,422 isolates.
