# Supplementary material for: Genetic Diversity of Meningococcal Serogroup B Vaccine Antigens among Carriage Isolates Collected from Students at Three Universities in the United States, 2015–2016
Source: mBio. 2021 May 18;12(3):e00855-21. doi: 10.1128/mBio.00855-21 (PMC8262942; doi:10.1128/mBio.00855-21)
Supplement: TABLE S5 [file mbio.00855-21-st005.pdf]

Table S5: Sequence typing profiles of *N. meningitidis* isolates recovered from repeat carriers who carried new strains during the carriage evaluation period.

Repeat carrier	Round	Vaccine doses[a]	Nm recovered	Genogroup[b]	MLST		Vaccine antigens			Fine typing	
					ST	CC	FHbp[c]	NadA	NhbA	PorA	FetA
1	1	0	Yes	B	409	CC41/44/ Lineage 3	A22/2.19	Not found	**p0002**	P1.18-1,34	F1-5
	2	N/P									
	3	N/P									
	4	1	Yes	*cnl*	823	CC198	B16/1.4	Not found	p0010	P1.18,25-14	Not found
2	1	0	Yes	*cnl*	2384	CC198	B16/1.4	Not found	p0010	P1.17,9	F5-5
	2	1	Yes	*cnl*	2384	CC198	B16/1.4	Not found	p0010	P1.17,9	F5-5
	3	N/P									
	4	2	Yes	C	269	CC269	B44/1.15	Not found	p0021	P1.17-1,23	F4-1
3	1	0	Yes	*cnl*	1136	CC1136	A103/3.94	Not found	p0145	P1.18-4,25-84	F3-6
	2	1	Yes	*cnl*	1136	CC1136	A103/3.94	Not found	p0145	P1.18-4,25-84	F3-6
	3	N/P									
	4	2	Yes	UD	11740	CC35	A106/2.27	Not found	p0021	P1.22-1,14	Not found
4	1	0	None								
	2	1	Yes	B	1130	CC32/ET-5	**B24/1.1**	NadA-1.1	p0003	P1.7-2,16	F3-3
	3	2	Yes	*cnl*	6321	unassigned CC for 6321	B16/1.4	Not found	p0010	P1.17,9	F5-5
	4	N/P									
5	1	N/P									
	2	0	Yes	*cnl*	823	CC198	B16/1.4	Not found	p0010	P1.18,25-14	Not found
	3	1	None								
	4	2	Yes	B	7574	CC41/44/ Lineage 3	B187/1.435	Not found	p0021	P1.22,14-6	F1-5
6	1	N/P									
	2	0	Yes	UD	35	CC35	A19/2.16	Not found	p0021	P1.22-1,14	F4-1
	3	N/P									
	4	1	Yes	*cnl*	12930	CC53	A73/2.102	Not found	p0058	P1.7;30-3	F1-2
7	1	N/P									

	2	N/P									
	3	0	Yes	E	178	CC178	B06/1.12	Not found	p0006	P1.18-1,9	F1-18
	4	1	Yes	E	11550	CC178	B06/1.12	Not found	p0006	P1.19-5,15-28	F5-2
8[d]	1		Yes	*cnl*	53	CC53	A73/2.102	Not found	p0024	P1.7-2,30-8	F1-2
	2		Yes	*cnl*	3085	CC35	A108/3.499	Not found	p0021	P1.7-2,13	F1-7
9	1	0	Yes	E	1157	CC1157	B09/1.13	NadA-2/3 frameshift	p0114	P1.21-7,16	F5-36
	2	1	Yes	E	1157	CC1157	B09/1.13	NadA-2/3 frameshift	p0114	P1.21-7,16	F5-36
	3	2	Yes	E	60	CC60	B09/1.13	Not found	p0024	P1.5,2	F1-7
	4	N/P									
10	1	0	Yes	E	60	CC60	B09/1.13	Not found	p0024	P1.5,2	F1-7
	2	1	Yes	E	1649	CC1157	B09/1.13	Incomplete ORF	p0010	P1.22,9	F5-36
	3	N/P									
	4	N/P									
11	1	0	Yes	E	1157	CC1157	B09/1.13	NadA-2/3 frameshift	p0114	P1.21-7,16	F5-36
	2	1	Yes	*cnl*	823	CC198	B16/1.4	Not found	p0010	P1.18,25-14	Not found
	3	2	None								
	4	N/P									
12	1	0	None								
	2	1	Yes	E	11739	CC60	B09/1.13	Not found	p0024	P1.5,2	F5-13
	3	2	Yes	UD	11740	CC35	A106/2.27	Not found	p0021	P1.22-1,14	Not found
	4	N/P									
13	1	0	None								
	2	1	Yes	E	60	CC60	B09/1.13	Not found	p0024	P1.5,2	F3-3
	3	2	None								
	4	3	Yes	B	2506	CC32/5	B30/1.224	NadA-1.100	p0003	P1.7,16-26	F3-3
14	1	0	Yes	*cnl*	34	CC32/5	**B24/1.1**	NadA-1.1	p1283	P1.19,15	F5-1
	2	N/P									
	3	N/P									

	4	1	Yes	B	409	CC41/44	A22/2.19	Not found	**p0002**	P1.18-1,34	F1-5
15	1	N/P									
	2	N/P									
	3	0	Yes	B	409	CC41/44	A22/2.19	Not found	**p0002**	P1.18-1,34	F1-226
	4	1	Yes	E	60	CC60	A15/2.25	Not found	p0024	P1.5,2	F1-7
16	1	0	Yes	*cnl*	823	CC198	B16/1.4	Not found	p0010	P1.18,25-15	F5-5
	2	1	Yes	*cnl*	12632	CC41/44	A22/2.19	Not found	**p0002**	P1.5-2,10-1	F1-5
	3	N/P									
	4	N/P									
17	1	0	Yes	E	60	CC60	B09/1.13	Not found	p0024	P1.5;2	F1-7
	2	1	Yes	UD	278	CC35	A12/2.24	Not found	p0021	P1.7-2;13-1	F1-7
	3	N/P									
	4	N/P									
18	1	0	Yes	B	11527	CC32/5	A32/2.101	Frameshift	p0237	P1.22,14-6	F3-15
	2	1	Yes	B	11527	CC32/5	A32/2.101	Frameshift	p0237	P1.22,14-6	F3-15
	3	N/P									
	4	2	Yes	B	35	CC35	A19/2.16	Not found	p0021	P1.22-1,14-31	F3-7
19	1	N/P									
	2	N/P									
	3	0	Yes	E	1649	CC1157	B09/1.13	Not found	p0010	P1.22,9	F5-5
	4	1	Yes	*cnl*	53	CC53	A73/2.102	Not found	p0058	P1.7,30-2	F1-2

Four cross-sectional carriage evaluations (rounds 1-4) were conducted at RI-1 and OR. Oropharyngeal swabs were collected from participants following vaccination. Two carriage evaluations were conducted at RI-2, where no outbreak or vaccine campaign occurred.

Abbreviations: UD, undetermined; *cnl*, capsule null locus; MLST, multilocus sequence typing; ST, sequence type; CC, clonal complex; Nm, *Neisseria meningitidis*.

Gray highlighted rows indicate the rounds at which vaccinated repeat carriers carried Nm and acquired a new strain. The carriage of Nm that is not gray highlighted shows that the Nm strain recovered at this round was the same as that in the next round.
[a] All 19 participants received MenB-FHbp (Trumenba); vaccine dose received at least 14 days prior to date of participation at the indicated round. N/P: not participated at the indicated round. Blank means no vaccine campaign occurred.
[b] Genogroup determined using whole-genome sequences.

[c] Both Pfizer (MenB-FHbp) and GSK (MenB-4C) nomenclatures are shown.
[d] Participant from RI-2 (no vaccine campaign occurred).
Boldface letter shows presence of the antigen peptide included in the MenB vaccines. Only antigen peptides included in MenB-4C were found among MenB-FHbp vaccinated repeat carriers shown in this table.
